# Kissing Lesions in Paederus Dermatitis

**DOI:** 10.4269/ajtmh.19-0109

**Published:** 2019-07

**Authors:** Palaniappan Vijayasankar, Hima Gopinath, Kaliaperumal Karthikeyan

**Affiliations:** Department of Dermatology, Venereology and Leprosy, Sri Manakula Vinayagar Medical College and Hospital, Pondicherry, India

**Figure 1.**
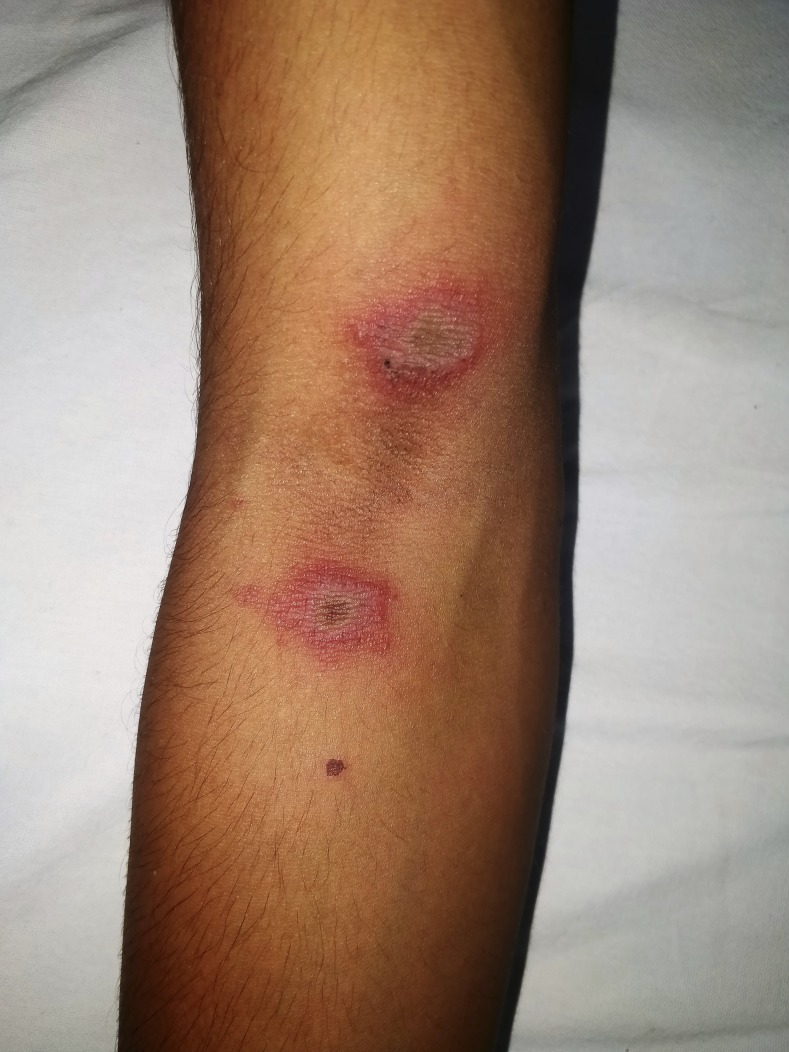
Two well-defined erythematous plaques with central cyanotic hue. This figure appears in color at www.ajtmh.org.

A 26-year-old south Indian laborer presented with two well-defined erythematous plaques with central cyanotic hue, equidistant from his right elbow crease on flexor aspect (Figure 1). The patient had associated burning sensation at the site of lesion. The patient slept outdoors the night before and noticed the lesions immediately after waking up in the morning. The lesions were typical of kissing lesions of paederus dermatitis that occurs because of the transfer of the irritant from one area to the adjacent areas of skin.^1^ The diagnosis of paederus dermatitis was made, which is common in this region. The patient was treated with topical betamethasone and fusidic acid. The lesions resolved in 2 weeks without residual pigmentation.

Paederus dermatitis is an acute irritant contact dermatitis caused by the accidental crushing of the *Paederus* beetle, which releases its coelomic fluid containing the potent vesicant paederin.^2^ Although paederus dermatitis is seen in all zoogeographic regions across the world, it is more common in humid tropical and subtropical regions with higher incidence during rainy season. The *Paederus* group of insects belongs to the Staphylinidae family, order Coleoptera.^3^ *Paederus* beetles are nocturnal in nature and draws itself to incandescent and fluorescent lights.^4^

On crushing the insect, there is erythema followed by vesiculation, crusting, and desquamation. The lesions are self-limiting. However, hyperpigmentation is a common sequela that may last for a month. Extensive exfoliation and ulceration can also occur. Apart from kissing lesions, other common morphological variants include dermatitis linearis and localized pustular dermatitis. There may be passive transfer of toxin to the genitals through fingers, producing lesions there. Similarly, ocular involvement also can occur, which may present as keratoconjuctivitis or periorbital dermatitis popularly known as “Nairobi Eye.” The lesions are usually noticed on awakening in the morning because of the nocturnal nature of the insects. It is thus termed as “night burn”or “wake and see” disease.^5^ Treatment of paederus dermatitis initially involves removing the irritant by washing the area with soap and water, and using wet compresses followed by the application of topical steroid.^4^
